# A 2-tier subdivision of papillary proliferations of the endometrium (PPE) only emphasizing the complexity of papillae precisely predicts the neoplastic risk and reflects the neoplasia-related molecular characteristics—a single-centered analysis of 207 cases

**DOI:** 10.1007/s00428-022-03367-8

**Published:** 2022-07-07

**Authors:** Danyang Liu, Tingting Chen, Kexuan Yu, Jing Li, Shunni Wang, Xiaoxi Ma, Qin Zhu, Yan Ning, Yiqin Wang

**Affiliations:** Department of Pathology, Obstetrics and Gynecology Hospital of Fudan University, Shanghai, 200090 China

**Keywords:** PPE, Endometrial carcinoma, *KRAS* mutation, Neoplasia

## Abstract

Papillary proliferation of the endometrium (PPE) is subdivided based on the complexity of the papillae and the proliferation of lesions, and the complex group is considered to have an increased risk of concurrent/subsequent endometrial neoplasia. However, the current subdivision criteria fail to prove the equivalence of the quantity of simple papillae and structural complexity. In this study, we divided PPE of 207 cases from 2014 to 2022 into 3 groups according to structural complexity and proliferation degrees: Group 1 equaled to the simple PPE with a simple papillary structure and typical localized proliferation; group 2 had the simple structure similar to group 1 but occupy over 50% of the endometrial polyp or > 2 lesions in the surface of nonpolypoid endometrium; group 3 had the truly complex branching papillae despite of its proportion. Group 3 was implicated with significantly more concurrent endometrial neoplasia (EAH and carcinoma) compared with groups 1 and 2 (*P* < 0.01), while no difference was found between groups 1 and 2. In 128 cases with no concurrent endometrial abnormalities in the initial biopsy or curettage specimens, 4 cases presented endometrial neoplasia (3 carcinoma and 1 atypical hyperplasia) in the subsequent specimens, all of which presented PPE of group 3 but not group 1 or 2 in the prior tissues (*P* < 0.01). The immunochemistry of 83 cases showed similar expressions of ER, PTEN, ARID1A, PTEN, p16, β-catenin, and p53 between PPE and the surrounding normal endometrium. Nearly 100% of PPE cases lost expressions of PR. A total of 2/83 cases showing PAX2 expression were all in the group 3 and correlated with endometrial neoplasia (2/17, 11.76%, *P* < 0.05). 76/83 (91.57%) of PPE lesions had *KRAS* mutations, and the distributions of which were similar among 3 groups. The frequency of mucinous metaplasia was significantly higher in the PPE lesions with *KRAS* mutations (72/74, 97.30%, *P* < 0.01). Group 3 showed higher frequency of single *KRAS* mutations compared with the combination of groups 1 and 2 (*P* < 0.01). Finally, the concordance of *KRAS* mutation profiles between PPE and endometrial neoplasia was significantly higher in group 3 than either group 1 or 2 (*P* < 0.01), while no difference was found between group 1 and 2. Thus, a new 2-tier subdivision system only emphasizing the complexity of papillae is recommended, which might precisely predict the risk of endometrial neoplasia and neoplasia-related molecular characteristics.

**Supplementary Information:**

The online version contains supplementary material available at 10.1007/s00428-022-03367-8.

## Introduction

Papillary proliferation of the endometrium (PPE) is characterized by the presence of papillary structures covered with benign endometrial glandular cells on the surface of the endometrium, or more usually, in the background of the endometrial polyp [1]. Based on the architectural complexity of the papillae and extent of proliferation, Ip et al. subdivided PPE into simple and complex groups [1]. Simple PPE is featured with the primary simple papillae and is considered benign, while the complex PPE is highly related to the risk of concurrent/subsequent endometrial neoplasia [1–3]. However, the PPE subdivision criteria proposed by Ip et al. have overlapping areas in both quality and quantity, which may lead to different diagnoses due to inconsistent observations among pathologists. More importantly, it remains unclear whether the accumulation of simple papillae and the complex architecture of papillae reflect the equal neoplastic risk of surrounding endometrium. Besides, it is difficult to judge the proliferation degree of papillae in the fragmented curettage specimens, which brings difficulties to the subdivision of PPE.

*KRAS* gene belongs to *RAS* gene family (*KRAS*, *NRAS*, *HRAS*), which encodes a guanylate-binding regulatory protein (GTP) binding protein involved in the EGFR signaling pathway [4]. *KRAS* mutations have been found to facilitate tumorigenesis in approximately 30% of all human cancers and 23% of the endometrial hyperplasia and the endometrioid carcinoma [5–9]. Interestingly, previous studies have reported the presence of *KRAS* mutations in PPE [10, 11] while the results seemed to be contradictory. Stewart et al. found *KRAS* mutations in PPE with complex branching papillae, but not in simple PPE [10]. On the contrary, Liu et al. detected the high frequencies of *KRAS* mutations in both two groups of PPE [11]. Besides, it is still not clear whether the *KRAS* mutations in PPE show any specific correlations with the hyperplastic degree and the neoplastic risk of the adjacent endometrium. In short, the value of *KRAS* mutation in PPE remains elusive.

In this study, we subdivided the PPE lesions of 207 cases into 3 groups based on the papillae complexity and proliferation degree described by Ip et al. and investigated the tumorigenic risk as well as the *KRAS* status among these groups. The aim of this study was to develop a novel subdivision system to precisely predict the concurrent or subsequent neoplastic risk of endometrium in the presence of PPE.

## Materials and methods

### Case selection

A total of 207 cases including 161 local and 46 consultation cases in the Obstetrics and Gynecology Hospital of Fudan University were collected in this study from January 2014 to January 2022. The clinical information, including patients’ age at diagnosis, presenting complete, relevant medical history, and follow-up information were obtained from the hospital electronic medical systems. All patients denied therapies that may influence the endometrial pathologies. The local Ethics Committee of Obstetrics and Gynecology Hospital of Fudan University approved the study design and informed consents were obtained from all participating patients.

### Pathological findings

All retrieved cases were re-evaluated by 2 experienced surgical pathologists (YQ. Wang and Y. Ning). The WHO classification criteria for female genital tract tumors in 2020 were used to diagnose endometrial hyperplasia and neoplasia including endometrial atypical hyperplasia (EAH) and adenocarcinoma. The presence of accompanied metaplasia changes (mucinous, squamous, ciliated cell and eosinophilic) within areas of PPE was recorded. Besides, the occurrence of endometrial polyps and the status of the adjacent endometrium were also assessed. PPE was divided into 3 groups based on the complexity of the papillae and degree of proliferation described by Ip et al. Group 1 equaled to the definition of simple PPE by Ip et al., which was comprised of those with simple papillae with short, predominantly non-branching stalks but occasional secondary branches were allowed. Besides, the localized proliferations restricted to 1 to 2 foci were also included, involving the surface of polyps or nonpolypoid endometrium. Groups 2 and 3 comprised the complex PPE defined by Ip et al., in which group 2 was consisted of those with simple papillae including more than 50% of the endometrial polyp or the regional proliferations of simple papillae extending with more than 2 foci involving the surface of nonpolypoid endometrium, while group 3 was comprised of those with truly complex or elongated papillae with secondary branches or those with diffuse intracystic proliferation. Cases either with nuclear atypia or with the branching papillae of any confluent and cribriform structures were excluded from the study as they might suggest malignancies with papillary forms.

### Immunohistochemistry

Immunohistochemistry was performed on the automatic immunostainer BondIII (M-211668 and M-212599, Leica, Germany). Incubation with PBS buffer was used as a negative control instead of primary antibody. Nuclear staining in glandular epithelium was considered positive for ARID1A, PAX2, MLH1, MSH2, MSH6, PMS2, ER-alpha, PR and Ki67. The p16 staining was classified as negative (no staining in the tumor cells), patchy staining (focal and discontinuous staining in the nucleus/nucleus-cytoplasm despite the staining intensity), and block staining (diffuse and continuous staining in the nucleus/nucleus-cytoplasm in 100% tumor cells). The p53 expression was considered mutant if more than 80% of tumor cells showed diffuse and continuous nuclear strong positive or all negative. Staining of 1–80% of nuclei with variable intensity of staining was considered wild type expression. The cytoplasmic staining was considered positive for PTEN. The staining of β-catenin was classified as membrane, cytoplasm, and nucleus (Table S1). The graphs were taken under the microscope (BX51, Olympus, Japan).

### Mutation analysis

The consecutive slides (7–10 per case) of the archival formalin-fixed, paraffin-embedded (FFPE) tissue sections within the recent 3 years were prepared and the areas of PPE lesions were manually marked and separated from each slides for genomic DNA extraction. QIAamp DNA Kit (Qiagen, Valencia, CA, USA) was used for extraction, and about 13 ng (3 ng/µL) FFPE tissues stored were required for 1 reaction. One other consecutive slide was prepared for the control H&E to identify the remaining PPE. Detection of *KRAS* mutations was performed on ABI7500 using the amplification refractory mutation system with the *KRAS* Mutation Detection Kit (Ref: 8.01.20102W006A, Amoy Diagnostics Co. Ltd., Xiamen, China). The cycling parameters for the PCR Protocol were 5 min at 95 °C, followed by 15 cycles at 95 °C for 25 s, 64 °C for 20 s, and 72 °C for 20 s, and the other 30 cycles for 93 °C for 25 s, 60 °C for 35 s, and 72 °C for 20 s (the fluorescence channels were FAM and HEX/VIC). Codons 12, 13, 59, 61, 117, and 146 of *KRAS* including 19 mutations were detected (Table S2).

### Statistical analysis

Data were analyzed using SPSS version 22.0 (SPSS, Chicago, IL, USA). The frequencies of concurrent and subsequent neoplasia among the groups of PPE were compared using spearman rank order analysis. The immunochemistry of PAX2 and the analyses of *KRAS* mutation among the groups of PPE were compared using Fisher’s exact test. A probability value (*P*) < 0.05 was considered statistically significant.

## Results

### Histological features in three groups of PPE

**Fig. 1:**
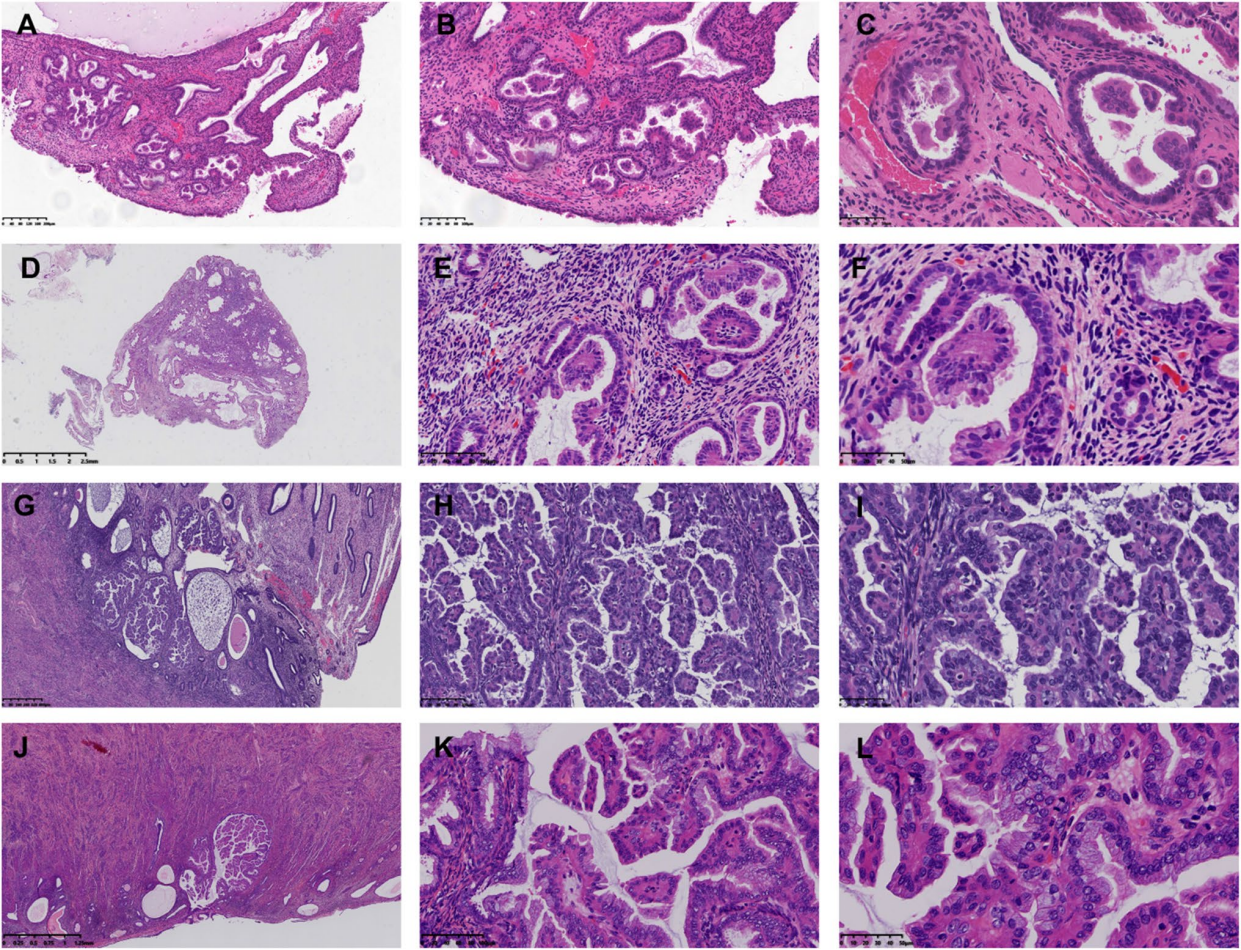
The histological features of 3 groups of papillary proliferations of endometrium (PPE). **A**–**C** The histological features of group 1. **A** The PPE lesions appeared in the background of the endometrial polyp. **B** The papillae were simple without any secondary branches. **C** Mucinous epithelium could be seen on the surface of papillae. **A** Scale bars = 200 µm. **B** Scale bars = 100 µm. **C** Scale bars = 50 µm. **D**–**F** The histological features of group 2. **D** The PPE lesions consist of simple papillae occupied over 50% of the endometrial polyp. **E**–**F** All the papillae had no complex branches and presented no cytological atypea. Mucinous metaplasia was conspicuous. **D** Scale bars = 2.5 mm. **E** Scale bars = 100 µm. **F** Scale bars = 50 µm. **G**–**L** The histological features of group 3 in the background of the endometrial polyp (**G**–**I**) and the nonpolypoid endometrium (**J**–**L**)**.** The group is characterized by the crowded intraluminal branching papillae with no cytological atypea. Still, mucinous metaplasia was conspicuous. **G** Scale bars = 400 µm. **H** Scale bars = 100 µm. **I** Scale bars = 50 µm. **J** Scale bars = 1.25 mm. **K** Scale bars = 100 µm. **L** Scale bars = 50 µm

A total of 207 cases ranged in age from 28 to 79 years old (average 55.27 ± 9.62 years old, median 56 years old); 65.07% were postmenopausal women. The cases consisted of 118 biopsy of polypectomy, 45 curettage, and 44 total hysterectomy (TH) specimens. Three consultation cases had TH surgeries due to the diagnosis of PPE. All the cases were classified into three groups: Group 1(122/207, 58.93%) equaled to the simple PPE described by Ip et al., which was characterized by less than 50% simple papillae or occasional detachment with no more than 2 foci in the surface of endometrium (Fig. 1A, B and C). Groups 2 and 3 were both belong to the definition of complex PPE defined by Ip et al., in which group 2 was simply the increment of simple papillae to more than 50% of the polyp with diffuse simple papillae (47/207, 22.71%) (Fig. 1D, E and F). Group 3 (38/207, 18.36%) was characterized by the truly crowded intraluminal branching papillae (Fig. 1G, H, I, J, K, and L) with no cytological atypia (Fig. 1I and L). About 64.25% of PPE (133/207) lesions were implicated with the background of the endometrial polyp (Fig. 1A, D, and G). Other 25 TH specimens (25/39, 64.10%) showed PPE in the nonpolypoid endometrium (Fig. 1J).

Almost 90.82% of PPE (188/207) presented with epithelial metaplastic alterations, the most common type of which was mucinous (173/207, 83.57%) (Fig. 1C, F, I, and L), followed by squamous (24/207, 11.59%), ciliated (22/207, 10.63%), and eosinophilic metaplasia (4/207, 1.93%). Only 15.46% (32/207) of the cases showed more than 1 type of epithelial metaplasia. Twenty-five mucinous metaplasia cases showed mucinous metaplastic alterations both in the PPE lesion and the surrounding endometrium. No goblet cells or extracellular mucin was identified.

### The incidence of concurrent and subsequent endometrial neoplasia in three groups of PPE

Forty-eight out of 207 cases were found concurrent endometrial abnormalities (23.19%) in the surrounding endometrium along with PPE in the initial specimens, 13 of which were from polyps, 13 from TH, and 22 from curettage tissues. Sixteen out of 48 cases were diagnosed with concurrent endometrial hyperplasia (16/48, 33.33%). Twenty-three out of 48 cases were EAH (47.92%) and 9 cases were adenocarcinoma (8 endometrioid and 1 undifferentiated subtype, 18.75%). The distribution of either concurrent EAH (*P* = 0.013) or carcinoma (*P* < 0.001) was statistically different in group 3 from the other 2 groups, and the overall incidence of neoplasia (EAH and carcinoma) was significantly elevated in group 3 (*P* < 0.01), while no difference was found between the other two groups (Table 1).

**Table 1:** Distributions of concurrent and subsequent endometrial abnormalities in three groups of PPE

PPE	Concurrent Endometrial status (*n* = 207)			*P*-value	Subsequent endometrial abnormalities (*n* = 128^a^)			*P*-value
	Normal	Hyperplasia	Neoplasia (EAH and carcinoma)		Normal	Hyperplasia	Neoplasia (EAH and carcinoma)	
Group 1	102	9	11	< 0.001*	75	0	0	< 0.001*
Group 2	36	5	6		35	1	0	
Group 3	21	2	15		13	0	4	
Total	159	16	32		123	1	4	
Group 1	102	9	11	0.328	75	0	0	0.261
Group 2	36	5	6		35	1	0	
Total	138	14	14		110	1	0	

*a* 128 cases showed no endometrial abnormalities in the initial biopsy or curettage specimens. * Spearman rank order. *P* < 0.05

The follow-up duration for 163 cases who had an initial endometrial biopsy or curettage was ranged from 1 to 88 months and the median follow-up was 27.5 months. Thirty-seven cases had subsequent TH, 8 of which were diagnosed as hyperplasia, 8 as EAH, and 2 as adenocarcinoma. The time intervals from the initial biopsy or curettage to the TH surgery in these 37 cases ranged from 7 days to 15 months, the median of which was 1 months. Two cases < 40 years were diagnosed as hyperplasia and then accepted the fertility-sparing therapy. They finally accepted the TH after 13 and 15 months. In the 128 cases with no endometrial abnormalities in the initial specimens, 4 cases found neoplasia (3 endometrioid carcinoma and 1 EAH) and 1 case showed complex hyperplasia in the subsequent TH specimens. All the 4 cases with neoplasia were located in the group 3, and the other 1 with complex hyperplasia was in group 2. The incidence of subsequent endometrial neoplasia in group 3 was significantly different from that either in group 1 or 2 (*P* < 0.01), while no difference was found between the other two groups (Table 1).

### The comparison of molecular characteristics among three groups of PPE

**Fig. 2:**
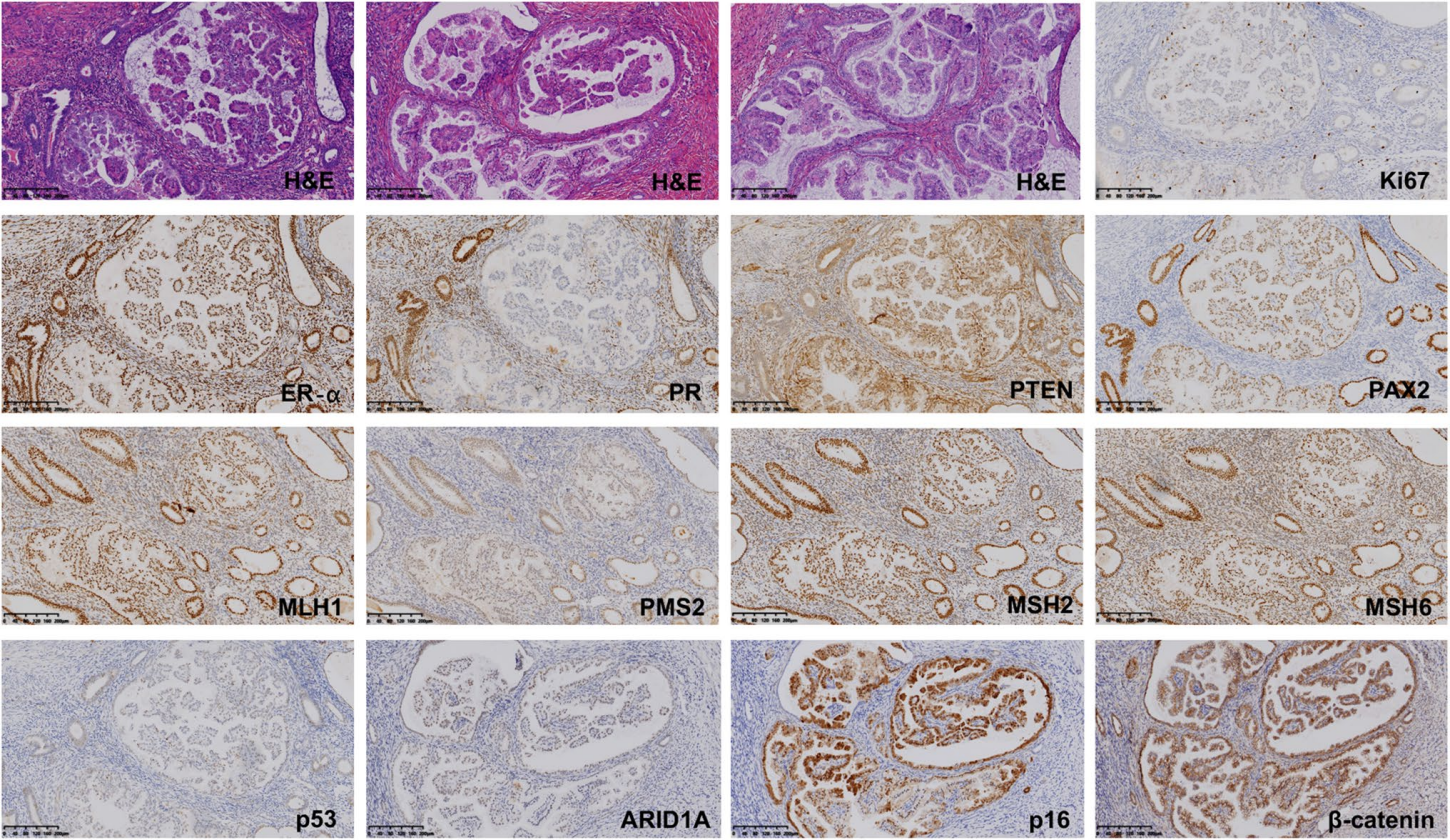
The immunochemical expressions of papillary proliferations of endometrium (PPE). The immunochemical patterns of MMR proteins (MLH1, MSH2, MSH6, PMS2), ER-α, PR, Ki67, PAX2, ARID1A, β-catenin, p16, PTEN, and p53 were illustrated. Scale bars = 200 µm

**Fig. 3:**
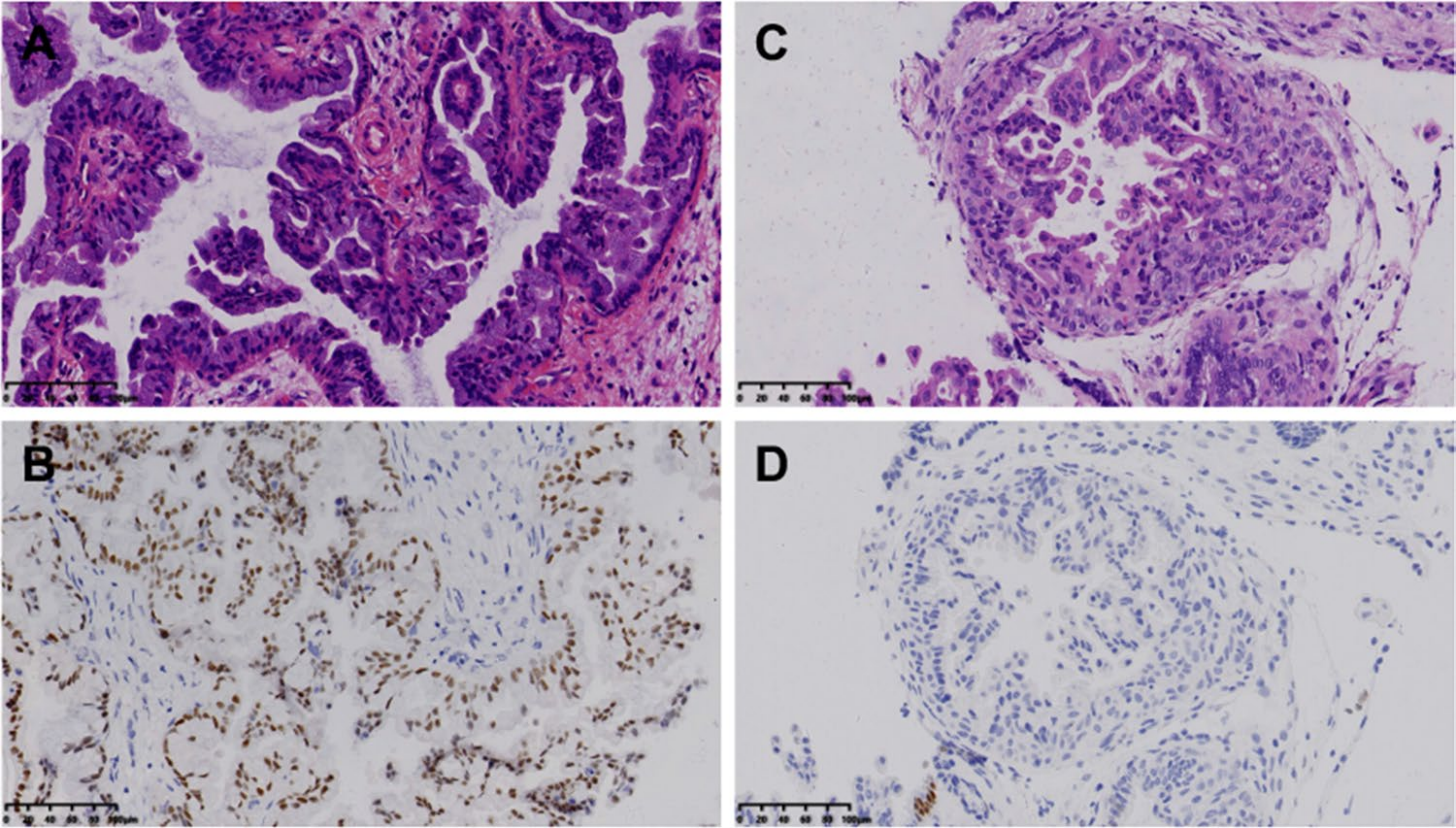
The representative graphs of different expressions of PAX2 in group 3. **A–B** The H&E (**A**) and representative positive PAX2 expression (**B**) in the group 3. **C**–**D** The H&E (**C**) and negative PAX2 expression (**D**) by immunochemistry cases in group 3. The complex branching papillae could be seen with mucinous metaplasia, and PAX2 expressions were lost with only individual positive cells. Scale bars = 100 µm

Because the detection of *KRAS* mutation should be performed on the FFPE tissues within 3 years, so, we picked 83 local cases from 2019 to 2022 for *KRAS* mutation analysis and immunochemistry. A total of 90.36% (75/83) cases were associated with predominant mucinous metaplasia. As shown in Fig. 2, all the PPE lesions in 83 cases showed positive expressions of MMR proteins, mild to moderate staining of ARID1A and PTEN and strong staining of ER-α and wild type of p53 expression. The Ki67 was low in all 83 cases despite the complexity of papillae. p16 showed the enhanced patchy staining in the PPE lesions while the surround normal endometrium presented equivalent expressions in the individual glands (Fig. 2). All the 83 cases presented membrane and ctyposmic staining of β-catenin (Fig. 2). PR expressions were lost in all 83 cases. A total of 81/83 cases showed weakened but retained nuclear staining of PAX2 in the PPE lesions (Figs. 2 and 3A–B), but 2 cases in the group 3 lost the expressions of PAX2 in the PPE areas (Fig. 3C, D), both of which had concurrent EAH. The proportion of negative PAX2 expression was higher in group 3 than either group 1or 2 (*P* < 0.05), while no difference was found between the other two groups (Table 2).

**Table 2:** The PAX2 expressions and *KRAS* mutations among groups of PPE

PPE	*N*	PAX2 IHC		*P* value	*KRAS* mutation		*P*-value	*KRAS* mutation type		*P*-value
		-	+		-	+		Single	Multiple	
Mucinous metaplasia	8	0	8	0.815	4	4	0.001*	4	0	0.331
Mucinous metaplasia +	75	2	73		3	72		54	18	
Group1	47	0	47	0.040*	5	42	0.662	30	12	0.036*
Group2	19	0	19		0	19		13	6	
Group3	17	2	15		2	15		15	0	
Group 1	47	0	47	–	5	42	0.172	30	12	0.519
Group 2	19	0	19		0	19		13	6	

^*^ Fisher’s exact test. *P* < 0.05

*KRAS* mutations were presented in 91.57% of cases (76/83), the incidence of which was significantly correlated with the presence of mucinous metaplasia (72/74, 97.30%, *P* < 0.01). The most frequent *KRAS* mutation was G12V (45/83, 54.22%), followed by G12D (29/76, 34.94%), G12A (8/83, 9.64%), G12C (5/83, 6.02%), Q61H and G13D (each 4/83, 4.82%), G12R (2/83, 2.41%), and G12S (1/83, 1.20%). No specific mutation profiles were found among three groups of PPE. A total of 58/76 (76.32%) *KRAS* mutations were single mutations, the frequency of which was significantly higher in group 3 when compared with the other 2 groups (*P* < 0.05, Table 2), while no difference was found between group 1 and 2.

### The comparison of *KRAS* mutation between groups of PPE and paired non-PPE endometrium

We further detected *KRAS* mutations in 71 surrounding endometrial tissues of PPE, which included 48 non-hyperplastic, 7 concurrent hyperplasia, and 16 concurrent neoplasia (11 EAH and 5 carcinoma) tissues. We also detected *KRAS* status in the subsequent carcinoma endometrial lesions of 2 cases, making a cohort of 73 paired non-PPE samples (Table 3). *KRAS* mutations were detected in 45/73 non-PPE samples (61.64%); the frequencies of which were significantly different among normal (43.18%), hyperplasia (87.50%), EAH (90.91%), and carcinoma lesions (55.56%, *P* < 0.01, Table 3). Although G12V was also the most frequent mutation in the non-PPE samples (26/45, 57.78%), only G12D showed statistically higher frequency in the neoplastic samples (EAH and carcinoma, 9/15, 60.00%) compared with normal (3/20, 15.00%) and hyperplasia samples (3/7, 42.86%, *P* < 0.05, Table 3). The concordance of *KRAS* mutation was higher between PPE and paired neoplasia samples (EAH and carcinoma, 13/18, 72.22%) compared with normal (16/48, 33.33%) and hyperplasia tissues (1/7, 14.29%, *P* < 0.01, Table 3).

**Table 3:** The *KRAS* Mutations in the paired non-PPE endometrial tissues

Non-PPE samples	*KRAS* mutation											
	mutation		*P*-value	Mutation type		*P*-value	Concordance with PPE		*P*-value	G12D		*P*-value
	-	+		Single	Multiple		*N*	*Y*		-	+	
Normal	25	23	0.002*	18	5	1.000	32	16	0.005*	20	3	0.007*
Hyperplasia	0	7		5	2		6	1		4	3	
Neoplasia (EAH and carcinoma)	3	15		11	4		5	13		6	9	
Total	28	45		34	11		43	30		30	15	

^*^ Fisher’s exact test. *P* < 0.05

Finally, the overall concordance of *KRAS* mutations between PPE lesions the paired non-PPE samples was significantly higher in group 3 (12/15, 80.00%) compared with either group 1 (10/40, 25.00%) or 2 (8/18, 44.44%) (*P* < 0.01, Table 4), while no statistical difference was found between group 1 and 2 (Table 4). Separated by the natures of paired non-PPE samples, the concordances of *KRAS* mutations between PPE lesions and paired normal or hyperplasia samples were similar among three groups (Table 4). In contrast, the concordance of *KRAS* mutations between PPE and paired neoplasia tissues was 91.67% in group 3, which was statistically higher than either group 1(1/4, 25.00%) or 2 (1/2, 50.00%) (*P* < 0.05, Table 4). No statistical difference was found between group 1 and 2 (Table 4).

**Table 4:** The concordance of *KRAS* mutations between PPE and non-PPE samples among 3 groups

Concordance of *KRAS* mutation	All non-PPE samples		*P*-value	Normal		*P*-value	Hyperplasia		*P*-value	Neoplasia (EAH and carcinoma)		*P*-value
	No	Yes		No	Yes		No	Yes		No	Yes	
Group 1	30	10	0.001*	23	9	0.470	4	0	0.429	3	1	0.022*
Group 2	10	8		8	6		1	1		1	1	
Group 3	3	12		1	1		1	0		1	11	
Total	43	30		32	16		6	1		5	13	
Group 1	30	10	0.121	23	9	0.259	4	0	0.333	3	1	0.600
Group 2	10	8		8	6		1	1		1	1	
Total	40	18		31	15		5	1		4	2	

* Fisher’s exact test. *P* < 0.05

## Discussion

This study might be the largest sample of PPE as we collected 207 cases diagnosed according to the criteria of Ip et al. from 2014 to 2022. We divided these cases into three groups by the quantity of simple papillae and complexity of branches. We found that compared with the groups with simple papillae of different quantities, the group 3 which had only complex branching architectures showed significantly higher frequencies of both concurrent and subsequent neoplasia of endometrium and also presented significantly more cases with loss of PAX2 expressions and more concordant single *KRAS* mutations occurring in both the PPE lesions and neoplasia endometrial tissues, while no difference was found between the other two groups. Therefore, we suggest a new 2-tier classification, which separates the group of complex branching papillae alone as the complex PPE, and the group with simple papillae regardless of the quantity as the simple PPE.

In 2013, Ip et al. first proposed a clear and integrated concept of PPE, defining it as a papillary change with no nuclear atypia, and proposed a 2-tier histological system by which the complex PPE conferred higher risk for endometrial neoplasia than the simple group [1]. The subsequent studies, however, found that the simple and complex PPE showed little difference considering the efficiency of alerting endometrial neoplasia [11], suggesting the possibility of unnecessary hysterectomy in some cases diagnosed with complex PPE as well as the defects of the current classification system. In our study, the frequency of concurrent neoplastic risk was elevated in the group with complex structures, while the quantification of simple papillae failed to show the difference. And only the group with complexity of papillae was able to suggest the presence of subsequent neoplastic risk. These results reflect the superior value of complex structure over quantification of simple papillae in terms of alerting the neoplasia. Thus, preserving the cases only with complex structures regardless of quantity in the complex PPE would predict more precisely the potential neoplastic risk of surrounding endometrium, making the category of PPE more valuable in clinical practice. Moreover, it would be now practical and feasible to evaluate the PPE lesions in the fractioned curettage specimens as the new classification only focusing on the structures of papillae, which would avoid the further overrated treatments.

Previous studies indicated that the immunochemistry of p16, PAX2, and β-catenin could be abnormal in the PPE lesions with different frequencies [10]. The diffuse positive nucleus of p16 could suggest the possibility of high grade carcinoma, and the nucleus staining of β-catenin as well as the loss of PAX2 suggest the risk of endometrioid neoplasia and poorer outcomes [10, 12]. In our study, p16 showed an enhanced but patchy staining in the PPE lesions, while β-catenin expressions were all located in the membrane and cytoplasm in the PPE, both of which were similar with the expressions in surrounding normal endometrium. It was interesting that all the cases lost the expression of PR in the PPE lesions, perhaps related to the overwhelming frequency of mucinous metaplasia in our study. The valuable marker was PAX2, the loss of which was seen in 2 case in the group 3 but not group 1 or 2, presenting a statistical discrimination. Moreover, both of these 2 cases had concurrent EAH, further supporting the correlation between complexity of papillae and neoplastic risk. Future studies should enlarge the sample for PAX2 detection ton confirm its value of relating PPE to the endometrial neoplastic risk.

The *KRAS* mutation is reported to exist in 10–30% of the type I estrogen-related endometrial cancer [9, 13] and predicts malignant transition as well as progression to advanced-stage disease[5, 6, 8]. Previous studies have found that complex PPE but not the simple group contains *KRAS* mutations [10], leading to the hypothesis that the complex group belongs to the spectrum of endometrial neoplasia. However, in our study, the frequencies of *KRAS* mutations were similar among three groups but were significantly correlated with the presence of mucinous metaplasia. Since *KRAS* mutation occurred in endometrial cancer was related to the mucinous metaplasia [12, 14–16], and Liu et al. also found that the incidence of *KRAS* mutation was higher in the PPE lesions with mucinous metaplasia; it was possible that the presence of *KRAS* mutation in PPE was determined by the mucinous differentiation [11] rather than the classification of PPE.

Recent studies using the whole-genome sequencing technique found that *KRAS* mutations exist not only in the endometrial cancer but also in the normal endometrium [17–19]. Lac et al. found that in a proportion of normal endometrium, the frequency of *KRAS* mutation (28%) was even higher than that in the endometrial cancer (19%) [20]. However, it was notable that the normal endometrial tissues always present the multiple mutations of *KRAS*, and the single mutation was considered as the real driven genetic variation in other genes [21]. In our study, we found that the frequency of single *KRAS* mutations was significantly higher in group 3, and the concordance of which with the paired neoplasia endometrial tissues was also elevated statistically in group 3, while no difference was found between the groups of different quantity of simple papillae, suggesting that the 2-tier classification emphasizing the complex papillae could reflect a distinctive *KRAS* characteristics of the complex PPE. Moreover, the detection of single *KRAS* mutations in the PPE with complex papillae could help with the evaluation of the neoplastic risk in the surrounding endometrium. More malignant samples complicated with PPE need to be recruited to corroborate this conclusion in future studies.

Finally, the *KRAS*^G12C^ inhibitor, AMG510, has been put into clinical application [24–27]. However, the *KRAS* mutations show uneven prevalence in the pan-cancer studies as *KRAS*^G12C^ and *KRAS*^G12V^ are predominant in non-small cell lung cancer (NCLC) while *KRAS*^G12R^ was more common in pancreatic ductal adenocarcinoma [4, 22, 23]. A previous study of all *RAS*-mutated tumors in the COSMIC database indicated that G12D followed by G12V mutations is the most frequent mutants in the endometrial cancer [23]. Similarly, our study found that both in the PPE and the surrounding non-PPE tissues, the most common *KRAS* mutations were G12V and G12D, and G12D showed a significant higher frequency in the neoplasia samples. Moreover, a recent study has reported that the hotspot mutations of *KRAS* could alter from G12C to G12V, G12D and G13D, etc. to escape the attack of the AMG510 and to develop putative resistance mechanisms exclusively [28]. In this case, a single hotspot mutant inhibitor might not effective in the endometrial cancer. Future studies could focus on the common downstream pathways of *KRAS* hotspot mutations in the endometrial neoplasia to explore additional therapeutic targets.

In summary, we found the new 2-tier system of PPE only emphasizing the complexity of papillae could better predict the potential risk of concurrent and subsequent endometrial neoplasia and reflect the neoplasia-related molecular characteristics, which could also help the pathologists to better interpret the presence of PPE, and to avoid overtreatments in these patients.

## Supplementary Information

Below is the link to the electronic supplementary material.

Supplementary file1 (DOCX 14254 KB)
