# Supplementary material for: A 2-tier subdivision of papillary proliferations of the endometrium (PPE) only emphasizing the complexity of papillae precisely predicts the neoplastic risk and reflects the neoplasia-related molecular characteristics—a single-centered analysis of 207 cases
Source: Virchows Arch. 2022 Jul 7;481(4):585–93. doi: 10.1007/s00428-022-03367-8 (PMC9534819; doi:10.1007/s00428-022-03367-8)
Supplement: Supplementary file 1 — Supplementary file1 (DOCX 14254 KB) [file 428_2022_3367_MOESM1_ESM.docx]

| Antibody | Source | Clone No. | Dilution |
| --- | --- | --- | --- |
| MLH1 | DAKO, Denmark | ES05 | 1:100 |
| MSH2 | DAKO, Denmark | FE11 | 1:200 |
| MSH6 | ZSJWBio, China | Ep49 | 1:200 |
| PMS2 | DAKO, Denmark | #1P51 | 1:1 ready to use |
| p53 | DAKO, Denmark | Do-7 | 1:300 |
| PTEN | DAKO, Denmark | 6H2.1 | 1:800 |
| p16 | SantaCruz, USA | JC8 | 1:1000 |
| ARID1A | Cell Signaling, USA | D2A8U | 1:1000 |
| β-catenin | BD Biosciences | Clone 14 | 1:1500 |
| Ki67 | DAKO, Denmark | MIB-1 | 1:200 |
| ER-alpha | Novocastra, UK | 6F11 | 1:50 |
| PR | Novocastra, UK | Clone 16 | 1:200 |

**Table S1. The information of antibodies used in the study**

**Table S2. The 19 mutations of the *KRAS* detected by the kit**

| Exon No. | *KRAS* Mutation | Base change |
| --- | --- | --- |
| Exon 2 | G12D | 35G>A |
|  | G12A | 35G>C |
|  | G12V | 35G>T |
|  | G12S | 34G>A |
|  | G12R | 34G>C |
|  | G12C | 34G>T |
|  | G13D | 38G>A |
|  | G13C | 37G>T |
| Exon 3 | Q61K | 181C>A |
|  | A59T | 175G>A |
|  | Q61R | 182A>C |
|  | Q61H | 183A>C |
|  | Q61H | 183A>T |
|  | Q61L | 182A>T |
| Exon 4 | K117N | 351A>C |
|  | K117N | 351A>T |
|  | A146T | 436G>A |
|  | A146V | 437C>T |
|  | A146P | 436G>C |
